# XPD suppresses cell proliferation and migration via miR-29a-3p-Mdm2/PDGF-B axis in HCC

**DOI:** 10.1186/s13578-018-0269-4

**Published:** 2019-01-05

**Authors:** Zhihua Xiao, Yijun Wang, Hao Ding

**Affiliations:** 1 Department of Gastroenterology, The Second Affiliated Hospital of Nanchang University, 1 Minde Road, Nanchang, 330006 Jiangxi People’s Republic of China; 2 The Second Clinical Medical College of Nanchang University, Nanchang, 330006 Jiangxi People’s Republic of China

**Keywords:** XPD, MiR-29a-3p, Cell proliferation and migration, Hepatocellular carcinoma

## Abstract

**Objective:**

The aim of this study was to investigate the role of XPD in migration and invasion of hepatocellular carcinoma (HCC) cells.

**Methods:**

The expression of XPD and miR-29a-3p was examined by western blot and qRT-PCR, cell proliferation was detected by MTT assay, cell migration was detected by transwell assay. TargetScan was used to predict potential targets of miR-29a-3p.

**Results:**

In this study, we found that the expression of XPD and miR-29a-3p was downregulated in HCC samples and HCC cell lines. XPD suppressed proliferation and migration of HCC cell via regulating miR-29a-3p expression. Target prediction analysis and dual-luciferase reporter assay confirmed Mdm2 and PDGF-B were direct targets of miR-29a-3p, and miR-29a-3p suppressed proliferation and migration of HCC cells via regulating the expression of Mdm2 or PDGF-B.

**Conclusions:**

Our data indicated that XPD suppressed cell proliferation and migration via miR-29a-3p-Mdm2/PDGF-B axis in HCC.

## Introduction

Hepatocellular carcinoma (HCC) is a primary neoplasm of the liver and the sixth most common solid tumor and the third most lethal malignancy globally [1]. Since effectively diagnosing HCC at its early stage is particularly difficult, only 20% of HCC patients are amenable to curative therapy by liver transplant, surgical resection, or ablative therapy, and even then, some of these patients suffer from the recurring tumors [2, 3]. Moreover, HCC commonly recurs after curative therapy, with the prognosis for HCC patients with advanced-stage disease remaining rather poor [1]. The need for novel therapeutic strategies is obvious and therefore, a better understanding of the underlying pathomechanisms is imperative.

Xeroderma pigmentosum D (XPD) is a subunit of transcription factor II H (TFIIH) [4], and involved in DNA unwinding during nucleotide excision repair (NER) [5]. In order to allow the damaged-specific nucleases to cleave the damaged DNA, XPD unwinds the DNA around the damaged site via stimulation of 5′ → 3′ helicase activity [6]. The liver is pivotal for many metabolic functions [7] and is very susceptible to carcinogenesis as the oxidant byproducts of hepatocellular metabolism often induced DNA damage. XPD has been reported to be down-regulated in patients with hepatocarcinoma [8]. Emerging evidence indicates that XPD could prime cell cycle arrest, induce HCC apoptosis and inhibit its viability [9], which implicated that XPD may reverse the malignant phenotype of hepatoma cells by repairing the damaged DNA. Here, we further investigated the influence of XPD on hepatoma cell proliferation in the molecular mechanism perspective.

MicroRNAs (miRNAs), as a class of small noncoding RNA 19–25 nucleotide in length, take part in negatively regulation of gene expression. MiRNAs have an essential influence on many fundamentally important biological processes including cell apoptosis, differentiation, proliferation and metabolism [10]. There is growing data have indicated that some tumor-specific miRNAs are widely downregulated or upregulated in HCC and closely associated with the occurrence and development of HCC [11, 12]. MiR-29a, currently one of the most interesting miRNA families in humans, has been shown to be silenced or downregulated in a wide range of cancers such as cell renal cell carcinoma [13], pediatric high-grade gliomas [14], in gastric cancer [15], including HCC [16].

We hypothesized that XPD might promote HCC cells migration and invasion through regulating the expression of miR-29a-3p. In this study, we first detected the expression of XPD and miR-29a-3p in tumor tissues from HCC patients. Furthermore, the underlying mechanism of XPD in the development of HCC was analyzed in vitro. This study might provide a better understanding of HCC pathogenesis and a potential therapeutic target for HCC intervention.

## Materials and methods

### Ethics statement

The study protocol was approved by the ethics committee of The Second Affiliated Hospital of Nanchang University, and all HCC patients provided written informed consents regarding the use of clinical specimens for the study.

### Sample collection and cell culture

Sixty-eight HCC tissue samples were collected from patients who underwent hepatectomy as treatment of HCC at The Second Affiliated Hospital of Nanchang University. Information pertaining to the clinicopathological parameters was also available. Liver cancer cell lines (HepG2, SMMC-7721 and Hep3B) were purchased from American Type Culture Collection (ATCC, USA) and the normal human hepatic cell line (LO2) was preserved in our laboratory and maintained in RPMI-1640 supplemented with 10% fetal bovine serum (FBS) (Hyclone, USA), 100 U/ml of penicillin (Gibco, USA), and 100 μg/ml of streptomycin (Gibco, USA) at 5% CO_2_ and 37 °C. The medium was changed every 2 days, and cells were passaged at 70–80% confluence.

### Cell transfection

The XPD overexpression plasmid, vector, miR-29a-3p inhibitor, inhibitor negative control (NC), miR-29a-3p mimic, mimic NC, Mdm2 overexpression plasmid or PDGF-B overexpression plasmid transfected into SMMC7721 cell. XPD siRNA, scramble, miR-29a-3p mimic or mimic NC, miR-29a-3p inhibitor or inhibitor NC, Mdm2 siRNA or PDGF-B siRNA were synthesized by GenePharma (Shanghai, China) and transfected into Hep3B cell. All cell transfections were introduced by Lipofectamine 2000 (Invitrogen Life Technologies, USA) according to the manufacturer’s instructions. For each cell transfection, three replicates were performed.

### Western blotting

Total proteins were extracted from Hep3B or SMMC-7721 cells using RIPA lysis buffer (Beyotime, China) and detected quantified with the BCA kit (Beyotime Biotechnology). Equal volume of protein were subjected to SDS-PAGE and transferred onto polyvinylidene difluoride membranes. After blocking in PBS with 5% skim milk for 1 h at room temperature, the membrane was incubated overnight at 4 °C with corresponding primary antibodies including XPD (1:1000; Abcam, Cambridge, UK), Mdm2 (1:100, Calbiochem, Bad Soden, Germany), P53 (1:400, Bioworld Technology Inc., Massachusetts, USA) and PDGF-B (1:1000; Abcam, Cambridge, UK), furthermore, it was incubated for 2 h with horseradish peroxidase (HRP) conjugated secondary antibodies at room temperature and the ECL kit was used to detect immunoreactive bands according to the manufacturer’s instructions (Thermo Scientific, Waltham, MA, USA).

### QRT-PCR

Total RNA was extracted from the transfected cells and frozen tissues using TRIzol reagent (Invitrogen, USA) following the manufacturer’s protocol. Reverse transcription was carried out using the High Capacity cDNA Reverse Transcription Kit (Applied Biosystems, Foster City, CA). cDNAs were subjected to real-time PCR with use of Power SYBR Green PCR Master Mix (Applied Biosystems) according to the manufacturer’s protocol. The results were calculated with the 2^−△△Ct^ method.

### Cell proliferation assay

The effect of XPD on SMMC7721 and Hep3B cell proliferation was measured by MTT assay. The cells were seeded in a 96-well plate at a density of 5000 monolayer cells per well. After 24 h, the cells were incubated with XPD for 24 h. Subsequently, the cells were washed with PBS and incubated with 20 µl MTT solution (5 g/l) for 4 h. After that, 150 µl DMSO (Shanghai Pharmaceutical Group, Shanghai, China) was added to each well to dissolve the crystals and then the plates were oscillated for 10 min in the dark. Finally, the optical density (OD) was measured at 490 nm using multifunctional fluorescence microplate reader. This experiment was performed in triplicate.

### Cell migration assay

Cell migration was assessed by Transwell assays. Cells were suspended in 100 μl serum-free medium and were plated in the upper chamber of each insert (Corning, USA) with a Matrigel-coated membrane (BD Bioscience, San Jose, USA). The lower chambers of the inserts were filled with DMEM medium with 10% FBS. After 24 h of incubation, cells that migrated to the lower surface of the insert were fixed, stained with 20% methanol and 0.2% crystal violet, and counted under a light microscope (Olympus, Tokyo, Japan).

### Luciferase reporter assay

Cells (5 × 10^4^ cells/well) were cultured in a 24-well plate and co-transfected with wild type (Mdm2-WT, PDGF-B-WT) or mutant (Mdm2-Mut, PDGF-B-Mut), miR-29a-3p mimic and mimic NC using Lipofectamine 2000 (Invitrogen) for 48 h. Firefly activity was normalized to luciferase reporter plasmid (pRL-CMV). Renilla activity as control of transfection efficiency. The luciferase activities were measured by the dual-luciferase reporter assay system (Promega, Madison, WI) according to the manufacturer’s instructions.

### Animal experiments

All animal experiments were approved by the Ethical Committee on Animal Experiments at the The Second Affiliated Hospital of Nanchang University. For tumor growth assays, SMMC7721 cells treated with lentiviral vector of XPD overexpression, miR-29a-3p antagomiR, XPD overexpression + miR-29a-3p antagomiR or vehicle were subcutaneously injected into the right scapulas of nude mice (5-week-old BALB/c-nude, 8 per group, 2.0 × 10^6^ cells for each mouse). The mice were observed over 34 days for tumor formation. The tumor volume was monitored every 3 days and calculated using the formula: V = 0.5 × length × width^2^.

### Statistical analysis

All date were analyzed with SPSS 16.0. Data were presented as mean ± standard deviation (SD). Student’s *t* test was used to analyze differences between two groups. One-way ANOVA analysis was used to determine the multi-sample analysis. Differences at *P *< 0.05 were considered to be statistically significant.

## Results

### The expressions of XPD and miR-29a-3p were downregulated in HCC

**Fig. 1:**
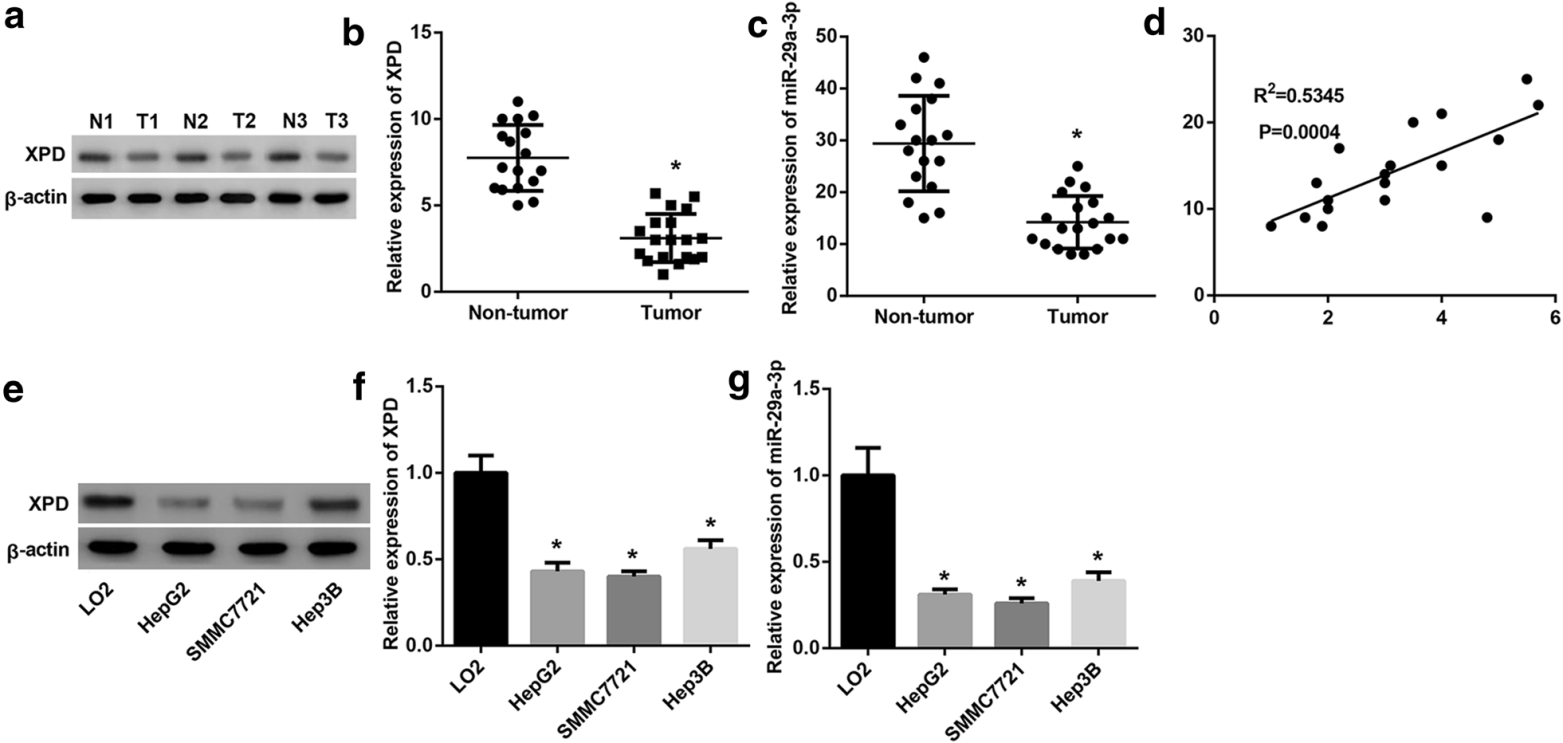
The expression of XPD and miR-29a-3p was downregulated in HCC. **a**, **b** Western blot and qRT-PCR analysis of XPD expression in HCC tissue samples and their corresponding adjacent non-tumorous liver samples. (n = 68). (*P < 0.05, vs. non-tumor). **c** QRT-PCR analysis of miR-29a-3p expression in HCC tissue samples and their corresponding adjacent non-tumorous liver samples. (n = 68). (*P < 0.05, vs. non-tumor). **d** Correlation analysis of miR-29a-3p and XPD expression HCC tissue samples. (n = 68). **e**, **f** Western blot and qRT-PCR analysis of XPD expression in normal human hepatic cell line (LO2) and HCC cells lines (HepG2, SMMC-7721, Hep3B). (*P < 0.05, vs. LO2). **g** QRT-PCR analysis of miR-29a-3p expression in normal human hepatic cell line (LO2) and HCC cells lines (HepG2, SMMC-7721, Hep3B). (*P < 0.05, vs. LO2)

To study the expression of XPD and miR-29a-3p in HCC, 68 paired HCC samples and adjacent non-tumor tissue samples were collected to examine the expression pattern of XPD and miR-29a-3p. The western blot and qRT-PCR results showed HCC samples exhibited lower levels of XPD expression as compared with non-tumor samples (Fig. 1a, b). Additionally, miR-29a-3p RNA level was also lower in tumor tissues than non-tumor tissues (Fig. 1c), and miR-29a-3p expression was positively associated with XPD expression in HCC samples (Fig. 1d). We further tested the XPD and miR-29a-3p expression in normal human hepatic cell line (LO2) and HCC cell lines (HepG2, SMMC-7721, Hep3B). The expression of XPD and miR-29a-3p was decreased in all the HCC cell lines when compared to LO2 (Fig. 1e–g). The above results implicated that XPD and miR-29a-3p might play a role in HCC tumorigenicity.

### XPD suppressed proliferation and migration of HCC cell via regulating miR-29a-3p expression

**Fig. 2:**
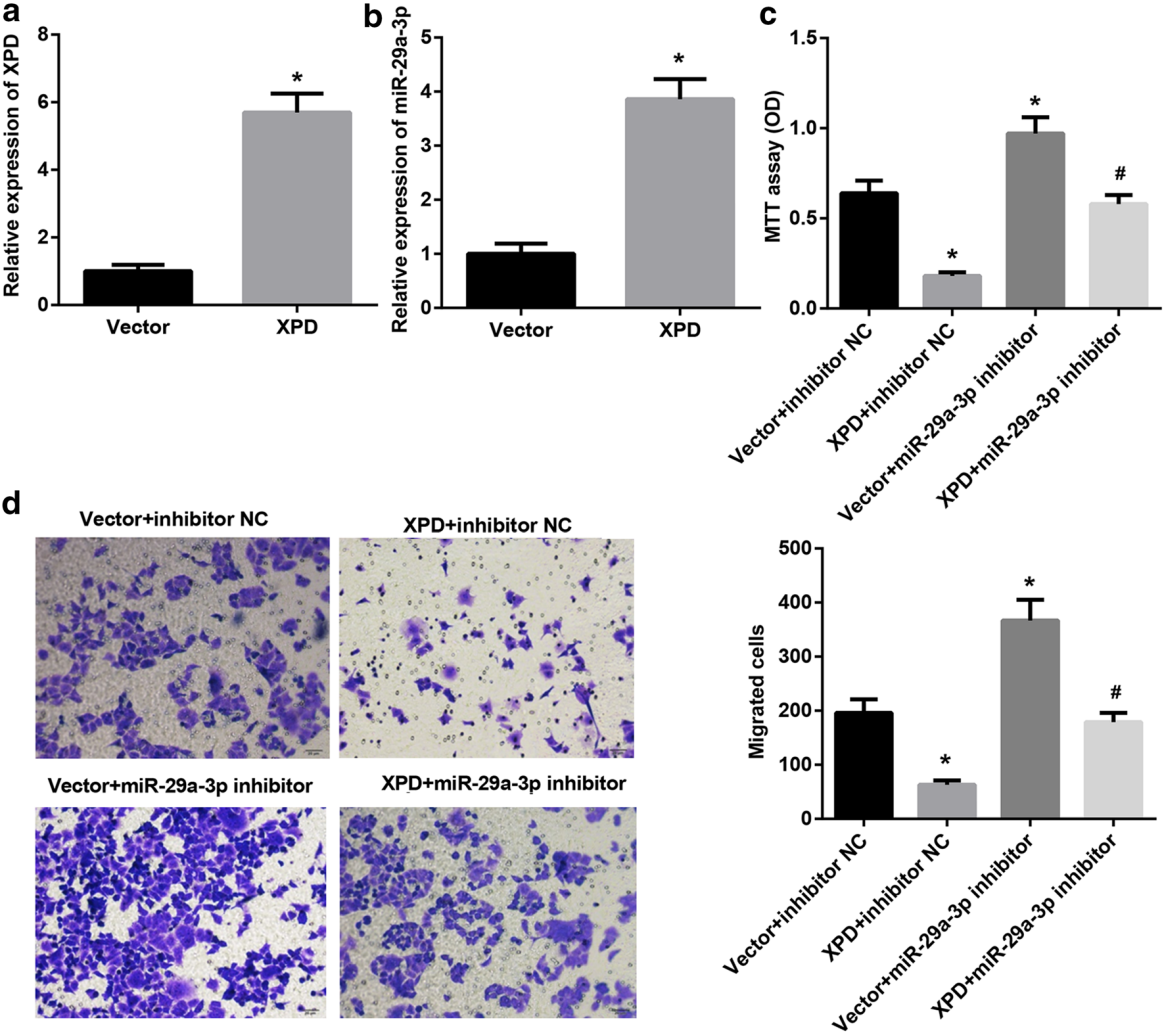
XPD suppressed proliferation and migration of SMMC7721 cell via regulating miR-29a-3p expression. **a**, **b** QRT-PCR analysis of XPD and miR-29a-3p expression in SMMC7721 cells after transfection with XPD overexpression plasmid. (*P < 0.05, vs. vector). **c** Proliferation ability test by MTT assay of SMMC7721 cells after transfection with XPD overexpression, vector, miR-29a-3p inhibitor or inhibitor negative control (NC). (*P < 0.05, vs. vector + inhibitor NC; ^#^P < 0.05, vs. vector + miR-29a-3p inhibitor). **d** Transwell migration assay of SMMC7721 cells after transfection with XPD overexpression, vector, miR-29a-3p inhibitor or inhibitor NC. (*P < 0.05, vs. vector + inhibitor NC; ^#^P < 0.05, vs. vector + miR-29a-3p inhibitor)

**Fig. 3:**
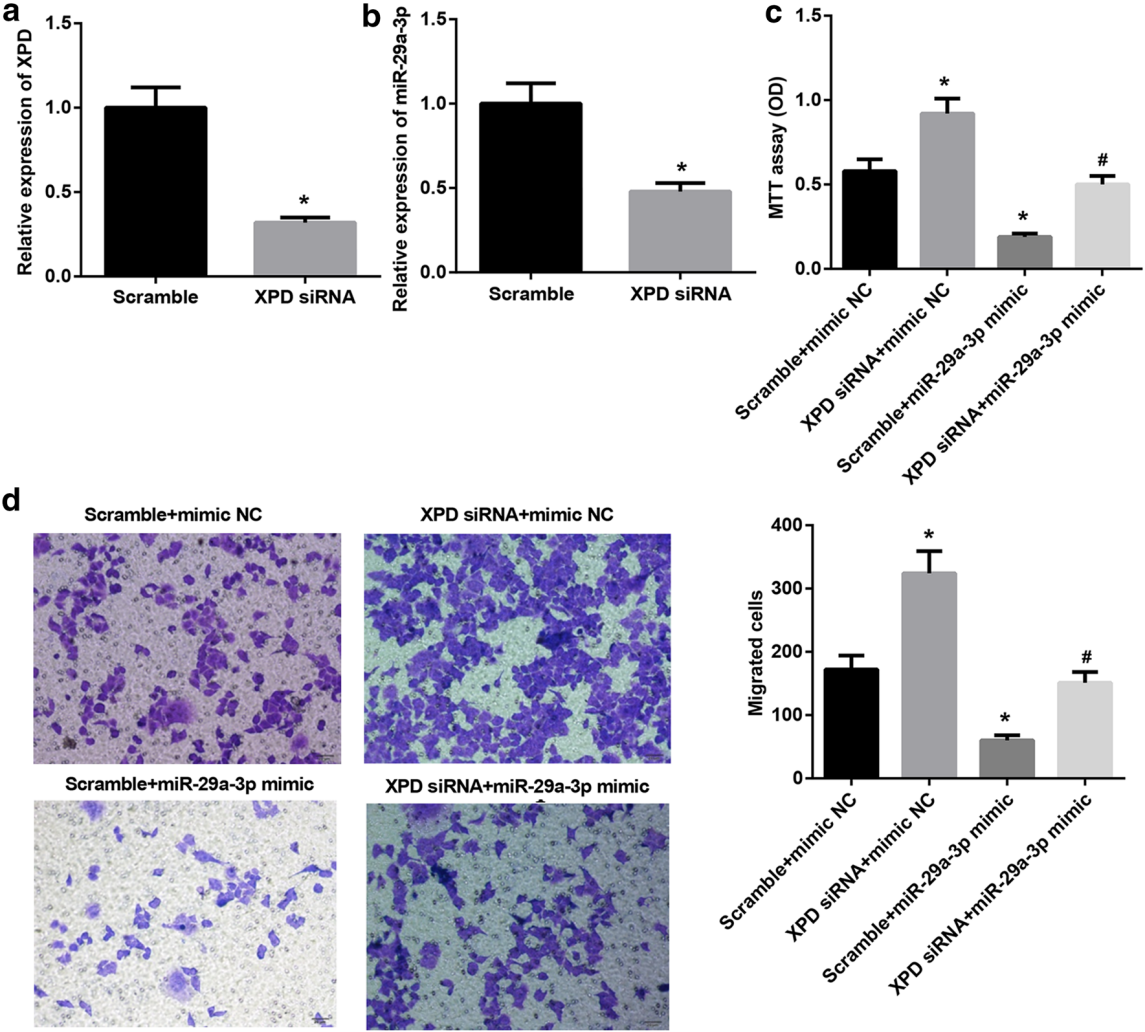
XPD suppressed proliferation and migration of Hep3B cell via regulating miR-29a-3p expression. **a**, **b** QRT-PCR analysis of XPD and miR-29a-3p expression in Hep3B cells after transfection with XPD siRNA or a scrambled non-targeting siRNA (scramble). (*P < 0.05, vs. scramble). **c** Proliferation ability test by MTT assay of Hep3B cells after transfection with XPD siRNA, scramble, miR-29a-3p mimic or mimic NC. (*P < 0.05, vs. scramble + mimic NC; ^#^P < 0.05, vs. scramble + miR-29a-3p mimic). **d** Transwell migration assay of Hep3B cells after transfection with XPD siRNA, scramble, miR-29a-3p mimic or mimic NC. (*P < 0.05, vs. scramble + mimic NC; ^#^P < 0.05, vs. scramble + miR-29a-3p mimic)

To investigate the effect of XPD and miR-29a-3p on cell proliferation and cell migration, the SMMC7721 and Hep3B were selected for further evaluation. SMMC7721 cells were transfected with XPD overexpression plasmid or vector control. The transfection efficiency of XPD overexpression plasmid was verified by qRT-PCR analysis (Fig. 2a). XPD overexpression significantly promoted miR-29a-3p expression in SMMC7721 cells (Fig. 2b). Then SMMC7721 cells were additionally transfected with miR-29a-3p inhibitor, MTT assay results indicated that miR-29a-3p inhibitor significantly promoted the cell proliferation of SMMC7721, and this proliferation could be reversed by XPD overexpression (Fig. 2c). Likewise, transwell assay data further revealed that miR-29a-3p inhibitor prominently promoted cell migration when XPD expression in SMMC7721 was enhanced (Fig. 2d). Then Hep3B cells were transfected with siRNAs targeting XPD or with a scrambled non-targeting siRNA as a negative control. Compared with control group, the expression of XPD and miR-29a-3p in XPD siRNA group was significantly reduced (Fig. 3a, b). Then Hep3B cells were additionally transfected with miR-29a-3p mimic, MTT assay and transwell assay results indicated that the ability of miR-29a-3p mimic to suppress proliferation and migration of Hep3B cell was markedly compromised when XPD expression was inhibited (Fig. 3c, d). From these results it is clear that XPD suppressed proliferation and migration of HCC cell via regulating miR-29a-3p expression.

### MiR-29a-3p directly targeted Mdm2 or PDGF-B

**Fig. 4:**
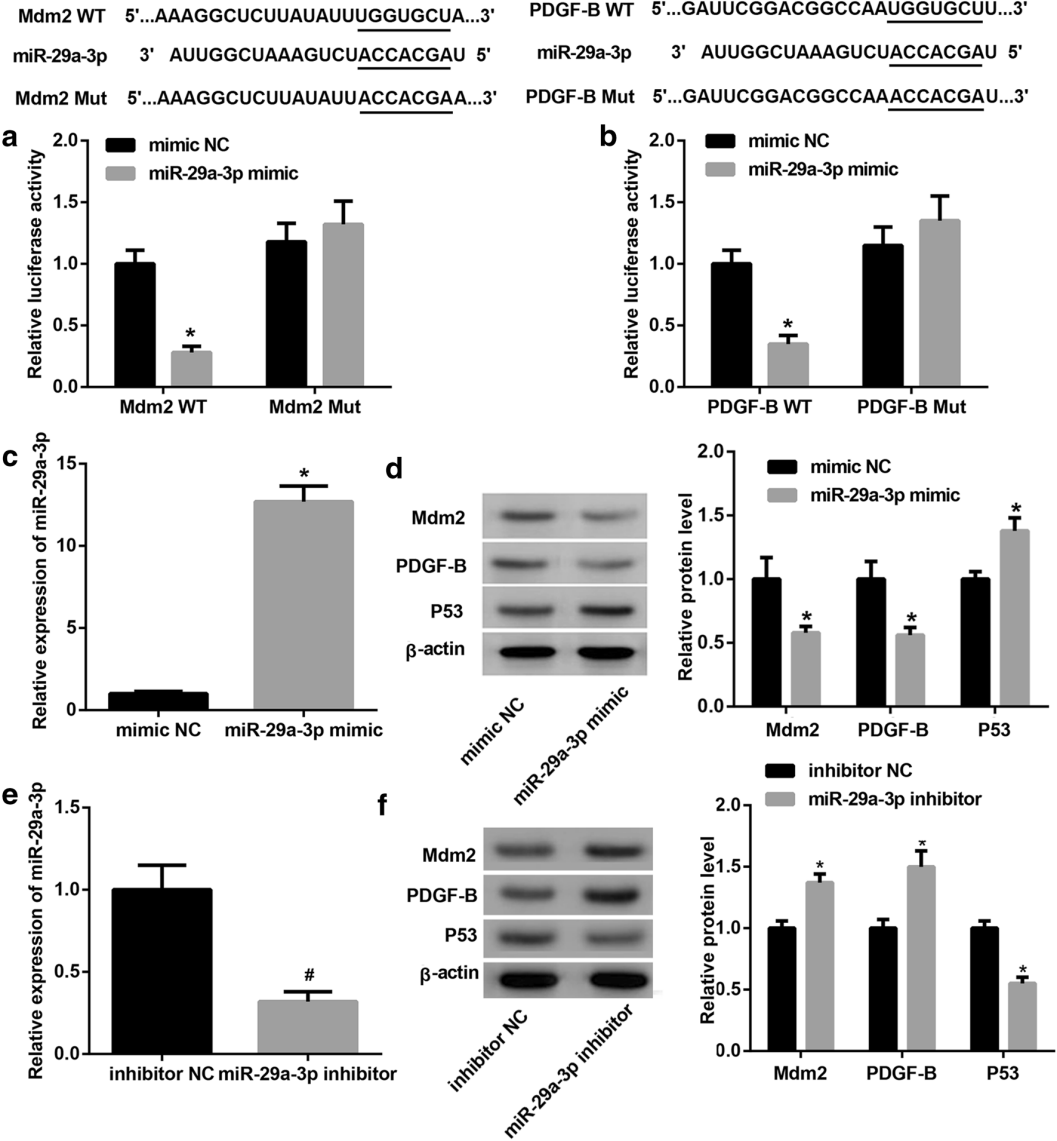
MiR-29a-3p directly targets Mdm2 or PDGF-B. **a** Schematic of the putative miR-29a-3p target site in Mdm2 3′-UTR and the seven mutated nucleotides are in lines. **b** Schematic of the putative miR-29a-3p target site in PDGF-B 3′-UTR and the seven mutated nucleotides are in lines. **c** QRT-PCR analysis of miR-29a-3p in SMMC7721 cells transfected with miR-29a-3p mimic or mimic NC. **d** Western blotting analysis of Mdm2, PDGF-B and P53 in SMMC7721 cells transfected with miR-29a-3p mimic or mimic NC. **e** QRT-PCR analysis of miR-29a-3p in Hep3B cells transfected with miR-29a-3p inhibitor or inhibitor NC. **f** Western blotting analysis of Mdm2, PDGF-B and P53 in Hep3B cells transfected with miR-29a-3p inhibitor or inhibitor NC. (*P < 0.05, vs. mimic NC; ^#^P < 0.05, vs. inhibitor NC)

To reveal the molecular mechanism that miR-29a-3p inhibited the proliferation and migration of HCC cell, miRNA target gene prediction site TargetScan was used to predict potential targets of miR-29a-3p. Among the candidates, we found highly conservative and specific combination sequence not only between miR-29a-3p and Mdm2 (Fig. 4a) but also between miR-29a-3p and PDGF-B (Fig. 4b). Our results showed that miR-29a-3p mimic significantly repressed luciferase activity when co-transfected with reporter containing WT Mdm2 3′UTR or WT PDGF-B 3′UTR but not MT Mdm2 3′UTRor MT PDGF-B 3′UTR (Fig. 4a, b). The miR-29a-3p mimic was transfected into SMMC7721 cells (Fig. 4c), the western blot result showed that miR-29a-3p mimic significantly suppressed protein expression of Mdm2 and PDGF-B while promoted P53 expression in SMMC7721 cells (Fig. 4d). Besides, when miR-29a-3p inhibitor was transfected into Hep3B cells, an opposite pattern is observed for Mdm2 and PDGF-B (Fig. 4e, f). Taken together, miR-29a-3p directly targets Mdm2 or PDGF-B.

### MiR-29a-3p suppressed proliferation and migration of HCC cells via regulating the expression of Mdm2 or PDGF-B

**Fig. 5:**
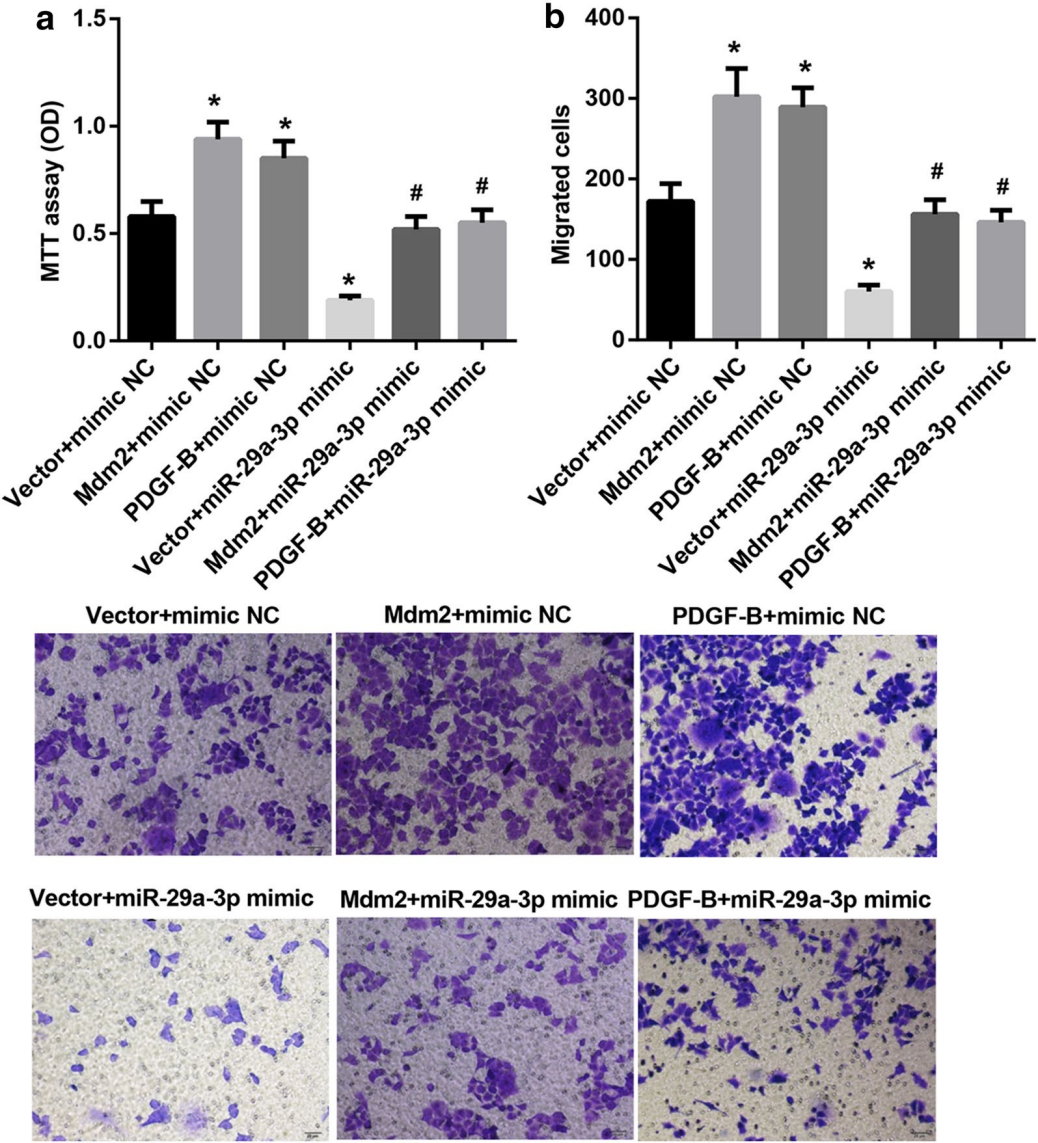
MiR-29a-3p suppressed proliferation and migration of SMMC7721 cells via regulating the expression of Mdm2 or PDGF-B. **a** Proliferation ability test by MTT assay of SMMC7721 cells after transfection with miR-29a-3p mimic and Mdm2 overexpression plasmid or PDGF-B overexpression plasmid. **b** Transwell migration assay of SMMC7721 cells after transfection with miR-29a-3p mimic and Mdm2 overexpression plasmid or PDGF-B overexpression plasmid. (*P < 0.05, vs. vector + mimic NC; ^#^P < 0.05, vs. vector + miR-29a-3p mimic)

**Fig. 6:**
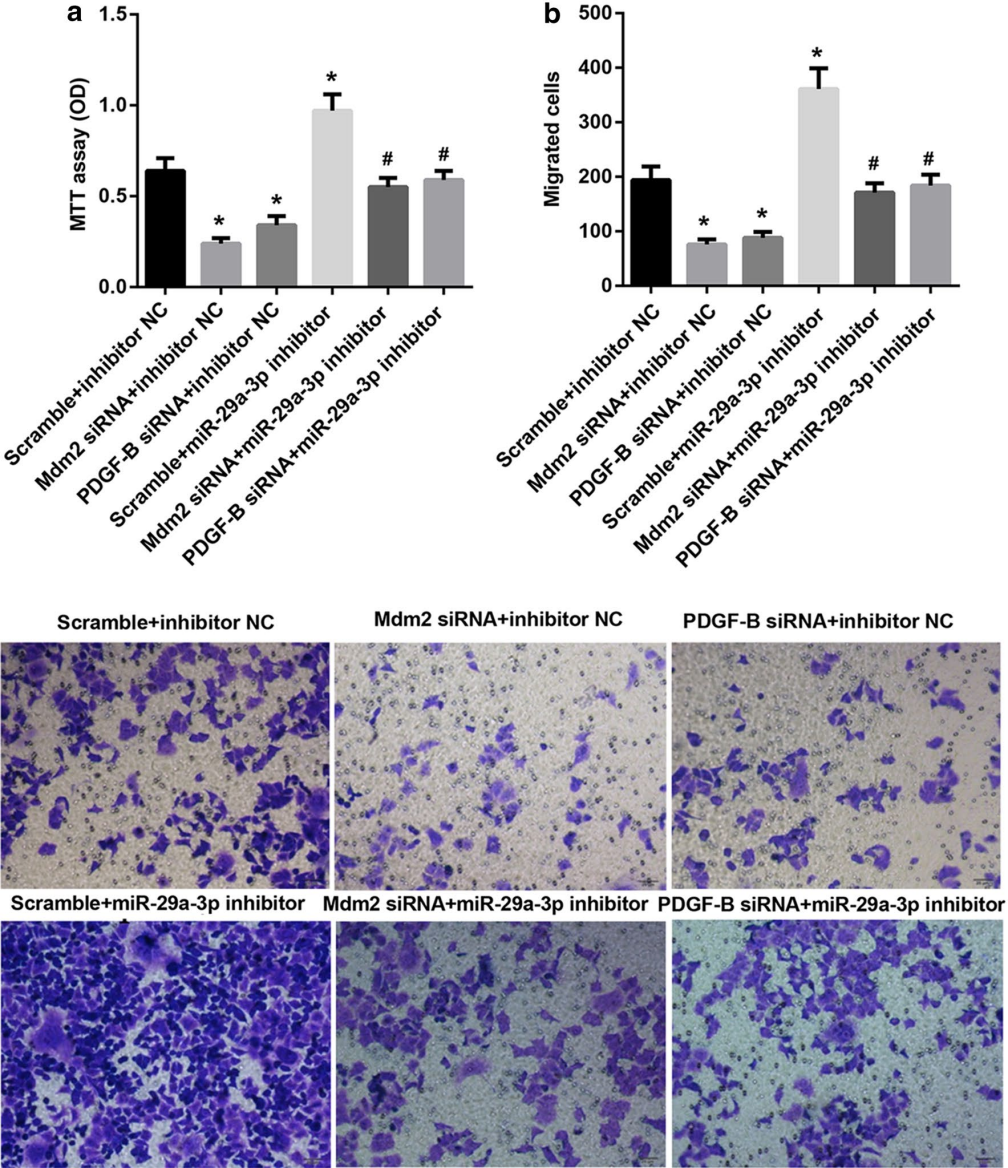
MiR-29a-3p suppressed proliferation and migration of Hep3B cells via regulating the expression of Mdm2 or PDGF-B. **a** Proliferation ability test by MTT assay of Hep3B cells after transfection with miR-29a-3p inhibitor and Mdm2 siRNAor PDGF-B siRNA. **b** Transwell migration assay of Hep3B cells after transfection with miR-29a-3p inhibitor and Mdm2 siRNA or PDGF-B siRNA. (*P < 0.05, vs. scramble + inhibitor NC; ^#^P < 0.05, vs. scramble + miR-29a-3p inhibitor)

To address whether miR-29a-3p can suppress cell proliferation and cell migration via regulating the expression of Mdm2 or PDGF-B, miR-29a-3p mimic and Mdm2 overexpression plasmid or PDGF-B overexpression plasmid were co-transfected into SMMC7721 cells. The results of MTT assay and transwell assay indicated that both Mdm2 and PDGF-B markedly promoted cell proliferation and cell migration in comparison with untreated group, moreover, miR-29a-3p remarkably repressed cell proliferation and cell migration, and this repression could be reversed by Mdm2 overexpression and PDGF-B overexpression (Fig. 5a, b). On the other hand, miR-29a-3p inhibitor and Mdm2 siRNA or PDGF-B siRNA were co-transfected into Hep3B cells. As seen in Fig. 6a, b, the ability of miR-29a-3p inhibitor to promote proliferation and migration of Hep3B cell was markedly reversed by Mdm2 siRNA or PDGF-B siRNA.

### XPD suppressed proliferation and migration of HCC cell via miR-29a-3p-Mdm2/PDGF-B axis

**Fig. 7:**
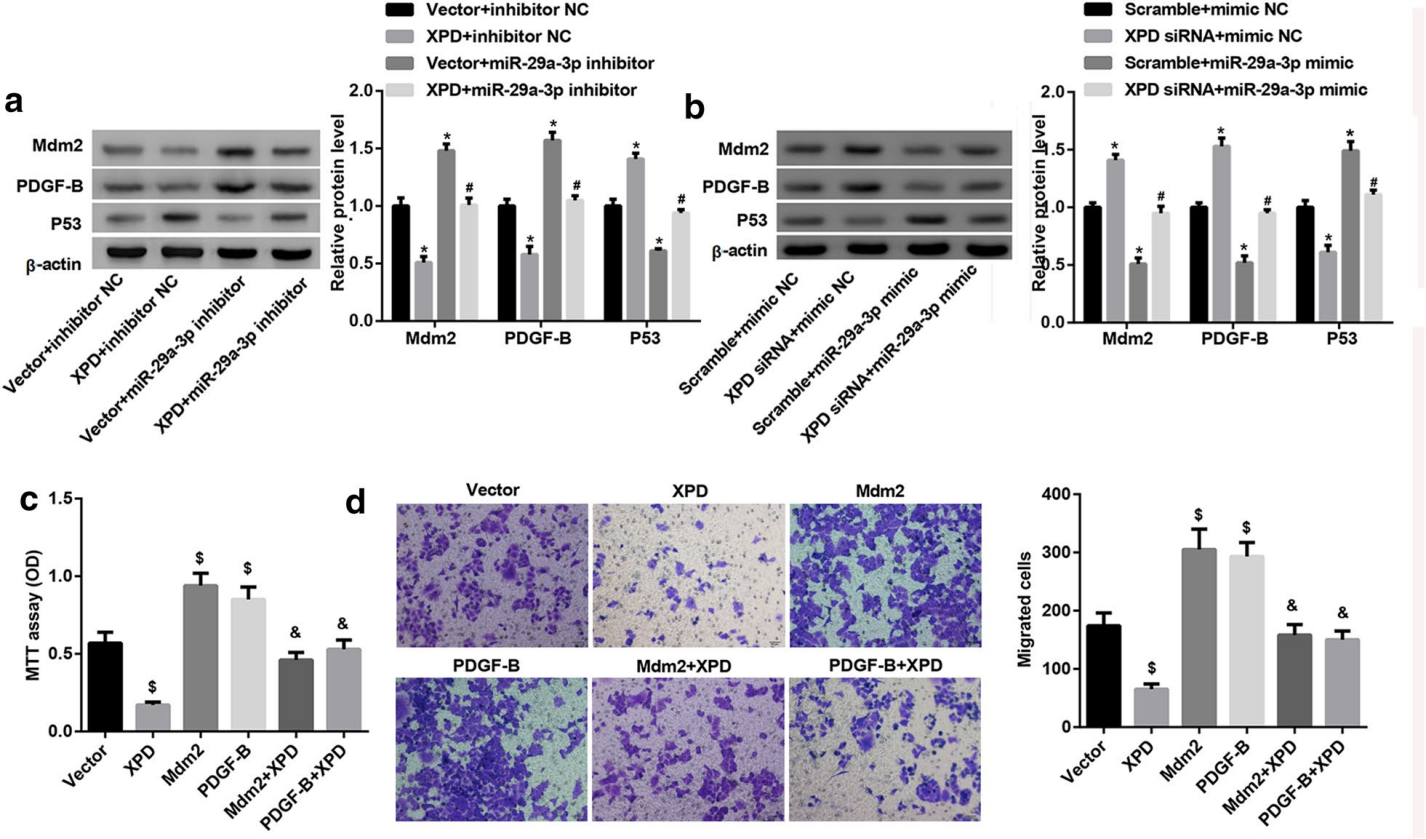
XPD suppressed proliferation and migration of HCC cell via miR-29a-3p-Mdm2/PDGF-B axis. **a** Western blotting analysis of Mdm2, P53 and PDGF-B expression in SMMC7721 cells after transfection with XPD overexpression plasmid and miR-29a-3p inhibitor. (*P < 0.05, vs. vector + inhibitor NC; ^#^P < 0.05, vs. vector + miR-29a-3p inhibitor). **b** Western blotting analysis of Mdm2, P53 and PDGF-B expression in Hep3B cells after transfection with XPD siRNA and miR-29a-3p mimic. (*P < 0.05, vs. scramble + mimic NC; ^#^P < 0.05, vs. scramble + miR-29a-3p mimic). **c** Proliferation ability test by MTT assay of SMMC7721 cells after transfection with XPD overexpression, Mdm2 overexpression plasmid or PDGF-B overexpression plasmid. (^$^P < 0.05, vs. vector; ^&^P < 0.05, vs. XPD). **d** Transwell migration assay of SMMC7721 cells after transfection with XPD overexpression, Mdm2 overexpression plasmid or PDGF-B overexpression plasmid. (^$^P < 0.05, vs. vector; ^&^P < 0.05, vs. XPD)

To further validated investigate the regulatory role of XPD on miR-29a-3p-Mdm2/PDGF-B axis, the SMMC7721 cells were transfected with XPD overexpression plasmid and miR-29a-3p inhibitor to examine Mdm2, P53 and PDGF-B expression by western blotting, the result in Fig. 7a showed that miR-29a-3p inhibitor induced protein levels of Mdm2 and PDGF-B were reversed in the presence of XPD, and an opposite pattern is observed for P53 in SMMC7721 cells. When XPD siRNA and miR-29a-3p mimic were co-transfected into Hep3B cells, the expression of Mdm2 and PDGF-B was enhanced while P53 expression was reduced as compared with scramble + miR-29a-3p mimic group (Fig. 7b). Furthermore, SMMC7721 cells were transfected with XPD overexpression, Mdm2 overexpression or PDGF-B overexpression, MTT assay results indicated that Mdm2 or PDGF-B blocked the ability of XPD to suppress proliferation and migration of SMMC7721 cell (Fig. 7c, d). Collectively, XPD suppressed proliferation and migration of HCC cell via miR-29a-3p-Mdm2/PDGF-B axis.

### XPD suppressed cancer cell growth in vivo

**Fig. 8:**
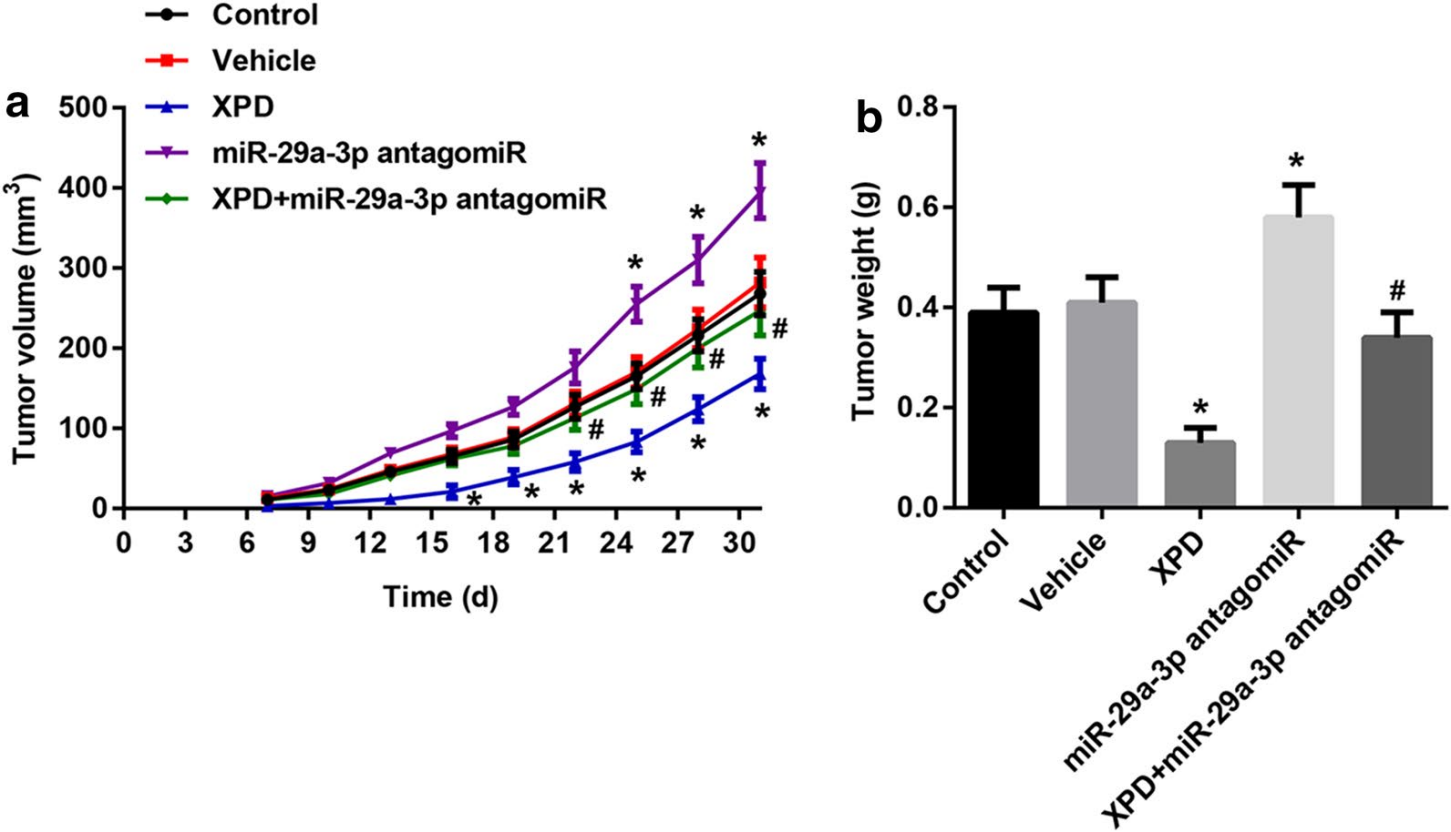
Functional test of XPD and miR-29a-3p in vivo and statistical results. SMMC7721 cells transfected with lentiviral vector (LV) of XPD overexpression, miR-29a-3p antagomiR, XPD overexpression + miR-29a-3p antagomiR or vehicle were injected subcutaneously into nude mice. **a** Tumor volume of nude mice from day 7 to day 34. **b** Tumor weight in each group. (^*^P < 0.05, vs. vehicle; ^#^P < 0.05, vs. miR-29a-3p antagomiR, n = 8)

To investigate the effects of XPD and miR-29a-3p on tumorigenesis in vivo, SMMC7721 cells transfected with lentiviral vector of XPD overexpression, miR-29a-3p antagomiR, XPD overexpression + miR-29a-3p antagomiR or vehicle were injected subcutaneously into nude mice to initiate tumor formation. 34 days later, large tumors were observed in the control and vehicle groups, the tumor volume was minimal in those mice transplanted with the XPD overexpression cells and was maximal in mice transplanted with the miR-29a-3p antagomiR cells, but reduced tumor volume was observed in the XPD overexpression + miR-29a-3p antagomiR group (Fig. 8a). At the end of the experiments, the tumors were isolated and weighed. Tumors from the nude mice transfected with XPD weighed significantly less while tumors from the nude mice transfected with miR-29a-3p antagomiR weighed more than both the control and vehicle mice, besides, tumors from the nude mice transfected with XPD overexpression + miR-29a-3p antagomiR weighed prominently less than only miR-29a-3p antagomiR mice (Fig. 8b). These results were in line with the antiproliferation function of XPD in vitro and indicated that XPD overexpression elicited a strong anti-tumor effect in HCC in vivo.

## Discussion

XPD, a DNA helicase with 5′-3′ polarity, has been shown to be associated with a wide range of malignancies [17–19]. With specific respect to HCC, recent studies have tied XPD to increased HCC susceptibility [20, 21]. XPD expression serves as a tumor suppressor in HCC [22]. To better understand XPD’s role in HCC, we investigated the in vitro cellular effects of XPD expression in HCC cells through transfection of the XPD gene into the HCC cell line SMMC7721 and Hep3B. We found that, relative to controls, XPD significantly inhibited HCC cell proliferation and migration. These combined findings indicate that XPD expression serves as a tumor suppressor in HCC cells in vitro, which is consistent with other previous in vitro studies on HCC cell lines [23]. Previous studies have shown that miR-29a may act as a potential suppressor miRNA [24, 25]. For example, miR-29a was downregulated in cervical squamous cell carcinoma tissues and was correlated with its progression by inhibiting cervical cancer cell migration and invasion [24]. In this study, we found that miR-29a-3p was positively correlated with XPD expression, and suppressed cell proliferation and migration of HCC cell lines, which in line with other works [16], moreover, the ability of miR-29a-3p to suppress cell proliferation and migration was markedly compromised when XPD expression was inhibited. These data indicated that XPD suppressed proliferation and migration of HCC cell via regulating miR-29a-3p expression, these results also implied that XPD might act as a tumor-suppressor whose downregulation contributed to the progression of HCC.

Tumor suppressor p53 plays a central role in preventing tumor formation. The levels and activity of p53 is under tight regulation to ensure its proper function. Murine double minute 2 (Mdm2), a p53 target gene, is an E3 ubiquitin ligase. Mdm2 is a key negative regulator of p53 protein, and forms an auto-regulatory feedback loop with p53 [26]. Mdm2 often has increased expression levels in a variety of human cancers and promotes cancer cell proliferation [27–29]. In this study, we identified Mdm2 as a direct target gene of miR-29a-3p using bioinformatic prediction, dual-luciferase reporter assay and western blot. We also showed that the overexpression of miR-29a-3p inhibited Mdm2 protein expression and elevated P53 expression, we further validated that miR-29a-3p suppressed proliferation and migration of HCC cells via regulating the expression of Mdm2. P53 enhances Mdm2 transcription through p53 specific response elements in the promoter region of Mdm2, thus forming an auto-regulatory feedback loop, which is critical to control the balance of p53 and Mdm2 [27]. MiR-29a is upregulated by p53, and miR-29a can successfully elevate the phosphorylation level of p53 by repression of Wip1, a phosphatase of p53 [30]. Richard Moore et al. [31] revealed these feedback regulatory pathways are closely interlinked with the core p53-MDM2 autoregulation in that Wip1 upregulates MDM2 via inhibiting its degradation. Given that, we speculated XPD suppressed proliferation and migration of HCC cell via miR-29a-p53-MDM2 network. On the other hand, platelet-derived growth factor (PDGF)-B is critical signaling molecules which strongly promote multiple processes of tumorigenesis tumor progression, through stimulating angiogenesis and proliferation of tumor cells [32]. Biological relevance of this signaling pathway has been demonstrated by therapeutic strategies targeting PDGF signaling and thereby inhibiting tumor growth [33]. Previous study has shown that microRNA-363 suppresses the proliferation of hepatocellular carcinoma cells and the expression of PDGF-B was suppressed after miR-363 transfection [34]. In this part of the study, we observed that the proliferation of HCC cells was slowed down by miR-29a-3p targeting PDGF-B. Our study may prompt a way to promote expression of miR-29a-3p and block MDM2/PDGF-B expression in HCC.

In conclusion, we demonstrated that XPD suppressed HCC cell proliferation and migration via regulating miR-29a-p53-MDM2/PDGF-B pathways, providing a new regulation mechanism of XPD expression in tumorigenesis. XPD-miR-29a-p53-MDM2/PDGF-B pathway may be a novel target for treatment of hepatocellular carcinoma.
